# Research Note: First report on the detection of necrotic enteritis (NE) B-like toxin in biological samples from NE-afflicted chickens using capture enzyme-linked immunosorbent assay

**DOI:** 10.1016/j.psj.2021.101190

**Published:** 2021-04-20

**Authors:** Kyung-Woo Lee, Hyun S. Lillehoj, Woohyun Kim, Inkyung Park, Charles Li, Mingmin Lu, Charles L. Hofacre

**Affiliations:** ⁎ Animal Biosciences and Biotechnology Laboratory, USDA-ARS, 10300 Baltimore Ave, Bldg. 1043, BARC-East, Beltsville, MD 20705, USA; † Southern Poultry Research Group, Inc., 1061 Hale Road, Watkinsville, GA 30677, USA; ‡ Department of Animal Science and Technology, Konkuk University, 120 Neungdong-ro, Gwangjin-gu, Seoul 05029, South Korea

**Keywords:** *Clostridium perfringens*, detection, ELISA, necrotic enteritis, NetB toxin, broiler chickens

## Abstract

Necrotic enteritis (**NE**) is a devastating enteric disease caused by *Clostridium perfringens* type G. One of the pore-forming toxins, NE B-like (**NetB**) toxin, secreted by pathogenic *C. perfringens* type G, has been proposed to be the main virulent factor in NE pathogenesis. The present study aimed to detect the presence of NetB toxin in biological samples of NE-afflicted chickens using NetB-specific monoclonal-based enzyme-linked immunosorbent assay (**ELISA**). Biological samples, including serum, digesta, and fecal droppings, were obtained from three previous NE studies (designated as Trials 1 to 3). In Trials 1 and 2, broiler chicks were infected with *Eimeria maxima* strain 41A on day 1 and followed by the *netB*-positive *C. perfringens* on day 18. Serum samples were obtained at 20 d post-hatch (i.e., 2 d post *C. perfringens* infection). In addition, various samples, including serum, gut digesta, and fecal droppings, that had been collected 0, 6, 24, and 30 h post *C. perfringens* infection were obtained. In Trial 3, broiler chicks were indirectly infected with litter-contaminated *E. maxima* on d 14 and followed by *netB*-positive *C. perfringens* via drinking water on days 18, 19, and 20. Serum samples and fecal droppings were obtained 21 d post-hatch (i.e., 1 d post last *C. perfringens* infection). The results showed that NetB toxin was not detected in serum samples in Trials 1 and 3. No NetB toxin was detected in all samples obtained before *C. perfringens* infection in Trial 2. Low but detectable amounts of NetB toxin were found in the serum samples obtained 6 h post *C. perfringens* infection in Trial 2. While NetB toxin in digesta and fecal droppings was detected 6 h post *C. perfringens* infection, its level plateaued 24 and 30 h post *C. perfringens* infection. In Trial 3, NetB toxin was detected in fecal droppings from the NE group, and its concentration ranged from 2.9 to 3.1 ng/g of wet feces. In Trial 2, NE-specific lesions were not seen 0 and 6 h post *C. perfringens* infection but exhibited lesions were moderate to severe 24 h post infection, leading to a moderate association (*r* = +0.527) between NE lesions and NetB toxin in the gut digesta. This is the first study to use NetB-specific monoclonal-based capture ELISA to determine and report the presence of native NetB toxin in biological samples from NE-induced chickens.

## INTRODUCTION

Necrotic enteritis (**NE**) is an enteric bacterial disease in chickens that is caused by *Clostridium perfringens* type G infection. Avian NE has gained much attention recently in the post antibiotic/anticoccidial agents' era as this infection has resulted in huge economic losses of 2 to 6 billion US dollars in the global poultry industry (Lee et al., 2020). Furthermore, the recently discovered NE B-like (**NetB**) toxin secreted by *C. perfringens* type G has been proposed to be the major toxin involved in the pathogenesis and development of NE in broiler chickens (Keyburn et al., 2010). NetB toxin is a 33-kDa secreted β-barrel pore-forming toxin that forms 1.6- to 1.8-nm pores in susceptible cells (Keyburn et al., 2008, 2010).

Since the discovery of NetB toxin in NE pathogenesis, molecular-based detection has been utilized to detect *netB*-positive *C. perfringens* isolates in NE-afflicted broiler flocks (Keyburn et al., 2008, 2010). In addition to the PCR-based molecular method targeting the *netB* gene in *C. perfringens* isolates, western blotting or cytotoxicity assays have been used to detect native NetB toxin in in vitro culture supernatants of *netB*-positive *C. perfringens* isolates (Keyburn et al., 2008). To our surprise, no attempts have been made to detect or quantify native NetB toxin present in biological or environmental samples of NE-afflicted chickens or flocks. Recently, we developed a monoclonal-based NetB-specific capture enzyme-linked immunosorbent assay (**ELISA**) and demonstrated that the developed ELISA can detect the in vitro production of native NetB toxin using *netB*-positive *C. perfringens* isolates (Lee et al., 2020). We further attempted to determine whether the developed capture ELISA could detect native NetB toxin present in the biological samples of NE-afflicted chickens (Lee et al., 2020). In this study, we applied the developed ELISA to biological samples, including serum, intestinal digesta, and fecal droppings, that had been collected from three NE studies that were reported previously and elsewhere (Park et al., 2008; Li et al., 2017; Hofacre et al., 2019).

## MATERIALS AND METHODS

### Biological Samples From NE-Afflicted Chickens

To address the question of whether the developed ELISA (Lee et al., 2020) could detect native NetB toxin in biological samples including serum, digesta, and fecal droppings that had been collected (Park et al., 2008; Li et al., 2017), were used in this study. In addition, we obtained serum and fecal samples from an NE trial in collaboration with Dr. Hofacre. For the purposes of convenience, we designated the samples collected from the NE studies that had been reported previously or elsewhere as Trials 1 to 3.

We first applied the developed ELISA to serum samples obtained from the NE-afflicted chickens in the *Eimeria maxima*/*C. perfringens* dual-challenge NE disease model (Trial 1)(Li et al., 2017). In brief, 4 groups were included: naïve uninfected controls, *E. maxima* infection, *C. perfringens* infection, and *E. maxima*/*C. perfringens* dual-infection groups. Forty-eight 1-day-old broiler chicks were provided with an antibiotic-free mash diet up to 14 d in an *Eimeria*-free facility. The chickens were orally infected with *Eimeria maxima* 41A (5.0 × 10^3^ oocysts/bird) on day 14, followed by *C. perfringens* Del-1 strain (1.0 × 10^9^ colony-forming units/bird) on d 18. On 20 d post-hatch (i.e., 2 d post *C. perfringens* infection), 5 birds per group were euthanized by cervical dislocation, and blood was collected via heart puncture. Serum samples were obtained by gentle centrifugation and stored at −20°C until use. All experiments were approved by the Institutional Animal Care and Use Committees of the Beltsville Agricultural Research Center (Protocol No 17-027). In addition, to see the kinetic patterns in the NetB toxin during NE pathogenesis, several samples, including serum, gut digesta, and fecal droppings, that had been collected elsewhere in the dual NE model (Trial 2) were used (Park et al., 2008). In brief, twenty-five 21-day-old broiler chickens were orally infected with *E. maxima* on d 21 followed by *C. perfringens* infection on day 25. Six birds on 0, 6, 24, and 30 h post *C. perfringens* infection were euthanized and blood, gut digesta and fecal droppings were sampled for lesion scores and NetB toxin quantification. All biological samples (i.e., serum samples and supernatants from saline-extracted gut and fecal droppings) had been stored at −70°C.

Finally, we also obtained serum and fecal dropping samples from an NE experiment that had been tested in Georgia (Trial 3). The *E. maxima*/*C. perfringens* dual-challenge NE model was reproduced as it was published elsewhere (Hofacre et al., 2019). In brief, day-old male broiler chicks (Ross 708) were raised in 8 cages. The coccidiosis challenge consisted of the day-old vaccination plus 4,000 oocysts of *E. maxima* per pen in 20 mL of water poured onto the litter around the feeders and drinkers on d 14. For the *C. perfringens* strain #6 challenge, feed and water were withdrawn for 2 to 3 h prior to administration. Approximately, 125 mL of the *C. perfringens* #6 (C.P.#6) 15-h culture at approximately 1 × 10^8^ CFU/mL were added to 75 mL of water, thoroughly mixed, and administered via the drinkers. This 200-mL volume was consumed within 30 min, at which time feed and water were returned to the pen. This dose was given on days 18, 19, and 20 to birds in the challenged groups. On d 21, 3 birds per pen (*n* = 3/pen, *n* = 12/treatment) were humanely euthanized by cervical disarticulation and sampled for blood collection. In addition, 4 fecal droppings (*n* = 4/treatment) per treatment were sampled on the same day. Serum and fecal samples were shipped overnight to the Animal Biosciences and Biotechnology Laboratory (ABBL) in Beltsville. This study was approved by the Southern Poultry Research Group Institute Animal Care and Use Committee (IACUC).

### Capture ELISA for Detection of NetB Toxin

The mAb-based NetB-specific capture ELISA was developed and reported elsewhere (Lee et al., 2020). In brief, NetB mAbs as a capture antibody was coated in carbonate buffer at 0.5 μg/well into 96-well microtiter plates overnight at 4°C. The plates were washed and blocked, and the 100 μL samples diluted in 0.1% BSA/PBS were added to the wells and incubated for 2 h at room temperature. The plates were washed with PBS-T, and 100 μL/well of peroxidase-conjugated NetB toxin mAbs (0.2 μg/mL) as the detection antibody were added. The plates were incubated for 30 min and then developed with the TMB substrate. The reaction was stopped by 2 M sulfuric acid (50 μL/well), and optical densities at 450 nm were measured with a microplate reader. The concentrations of NetB protein in the biological samples were calculated from the standard curve generated with the known concentration of *Escherichia coli*-expressed NetB protein.

The developed ELISA has a Limit of Detection of 0.02 ng/mL and Limit of Quantitation of 0.14 ng/mL from the standard curve generated with a range of 200 ng to 2 pg NetB/mL (Lee et al., 2020). The ranges of intra- and interassay coefficients of variability were 1.31 to 8.40% and 1.07 to 8.23%.

### Statistical Analysis

All statistical comparisons were performed in GraphPad Prism version 5.0 (GraphPad Software, Inc., La Jolla, CA). The Tukey HSD test was used to disclose the difference between the mean values of the treatment groups at *P* < 0.05. The correlation coefficient between NE lesions and NetB protein in the gut digesta samples was calculated using Microsoft Excel 2016 (Microsoft Corporation, Redmond, WA).

## RESULTS AND DISCUSSION

**Figure 1:**
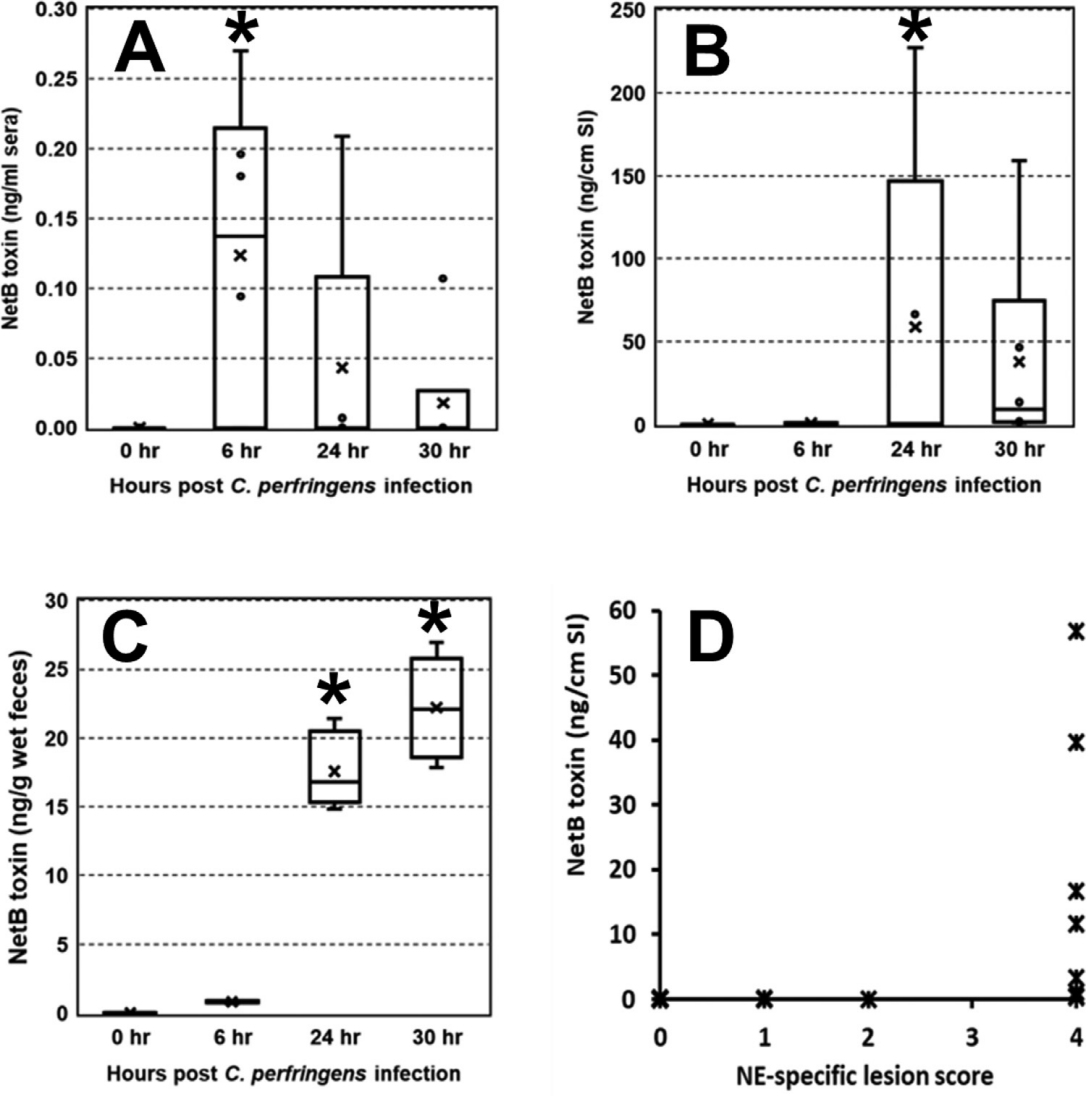
NetB toxin levels in sera (panel A), gut digesta (panel B), and feces (panel C) sampled post *C. perfringens* infection. All samples were obtained from NE-afflicted chickens reported elsewhere (Park et al., 2008). Correlation between NetB toxin in gut digesta and NE lesion score was depicted (panel D). Asterisk indicates significant increase over the initial value (day of *C. perfringens* infection) at *P* < 0.05. Correlation coefficient between NE lesions and NetB toxins was calculated to be r = 0.527.

No NetB toxin was detected in all serum samples obtained 2 d post *C. perfringens* infection from Trial 1 (Table 1). Next, we applied ELISA to several samples of blood, gut digesta, and fecal droppings obtained 0, 6, 24, and 30 h post *C. perfringens* infection (Trial 2). On the day of *C. perfringens* infection, none of the biological samples showed detectable NetB toxin (Figure 1A–1C). However, NetB toxin was detected in serum samples obtained 6 h or later post *C. perfringens* infection (Figure 1A–1C). Also, 6 out of 17 broilers showed moderate amounts of NetB toxin in the digesta samples, and its concentration was 0.6 ng per g 6 h post *C. perfringens*, which plateaued to 73.5 ng per g of sample 24 h post *C. perfringens* infection (Figure 1B). NetB toxin was found in all fecal droppings, and its concentration increased from 0.8 to 73.5 ng per g of wet feces (Figure 1C). The NE-specific lesions were not seen 0 and 6 h post *C. perfringens* infection. However, 5 NE broilers exhibited moderate to severe lesions with on average 2.4 (data not shown). At 30 h postinfection, all NE birds exhibited clinical NE lesions with a classic Turkish towel-like appearance. There was a moderate association (*r* = +0.527) between the NE lesion and NetB toxin in the gut digesta (Figure 1D). In Trial 3 (serum and fecal samples from Georgia), among 8 fecal samples, 3 fecal samples had detectable NetB toxin ranging from 2.9 to 3.1 ng per g of wet feces (Table 1). No NetB toxin was found in the serum samples.

**Table 1:** Detection of necrotic enteritis (NE) B-like (NetB) toxin in biological samples obtained from NE-induced chickens.

Trial	Treatment^1^	Samples type	Number sampled	No. positive^2^	NetB toxin, ng/g feces, or mL serum (SD^3^)	Reference
1	CONT	Serum	5	0	nd^3^	Li et al., 2017
	EM	Serum	5	0	nd	
	CP	Serum	5	0	nd	
	EM/CP	Serum	5	0	nd	
3	EM/CP	Serum	12	0	nd	Hofacre et al., 2019
		Feces	4	1	2.87	
	EM/CP+BMD	Serum	12	0	nd	
		Feces	4	2	3.09 (3.30)	

1 CONT = non-challenged naïve control, EM = *Eimeria maxima* challenged group, CP = *Clostridium perfringens* challenged group, EM/CP = *E. maxima* and *C. perfringens* dual challenged group, EM/CP+BMD = EM/CP plus antibiotic bacitracin methylene disalicylate in diet.

2 Samples exhibiting equal to or greater than detection limit (0.02 ng/mL) were considered positive.

3 SD = Standard deviation, nd = not detected.

Avian NE is a devastating enteric disease in chickens caused by *C. perfringens* type G. Avian NE is exhibited as either subclinical or clinical forms of infection depending on the severity of clinical signs. Historically, NE was closely linked to the capability of *C. perfringens* in producing dominantly alpha-toxin, which is a 370-amino acid necrotizing zinc metalloenzyme with phospholipase C and sphingomyelinase activity. However, the role of alpha toxin in NE pathogenesis has been questioned as alpha toxin-defective *C. perfringens* has been found to be able to reproduce experimental NE in chickens, which led to the discovery of NetB toxin as the virulent factor in NE (Keyburn et al., 2008). Until now, the presence of native NetB toxin was only confirmed in *in vitro* culture supernatants from *netB*-positive *C. perfringens* isolated from NE-afflicted or healthy chickens by western blot analysis using polyclonal antirecombinant NetB antiserum (Keyburn et al., 2008, 2010). However, the presence of NetB toxin in biological samples has not been reported, which might hinder the determination of the role of NetB in NE development. Recently, we developed NetB-specific monoclonal-based capture ELISA that can quantify the in vitro production of NetB toxin in *C. perfringens* culture supernatants (Lee et al., 2020). The developed ELISA has the advantages over the standard western blot assay including a more rapid analysis, lower cost, and the ability to test many samples in a single experiment. The developed ELSIA (Lee et al., 2020) has not been compared to other standard methods including western blot assay to determine sensitivity and accuracy of the assay.

We further attempted to use the developed capture ELISA to detect native NetB toxin in biological samples, including serum samples, gut digesta, and fecal droppings. To this end, we reproduced NE using a dual-infection model with *E. maxima* followed by *netB*-positive *C. perfringens* and sampled accordingly during the 0 to 30 h (Trial 2) or 2 d (Trial 1) post *C. perfringens* infection. In addition, serum samples and fecal droppings (Trial 3) were provided from NE-induced broiler chickens (see NE model in Hofacre et al., 2019) in collaboration with Dr. Hofacre. No detectable NetB toxin was found in serum samples on the day of (Trial 2), at 1 d (Trial 3), and 2 d (Trial 2) post *C. perfringens* infection. However, low but detectable NetB toxin as detected by capture ELISA were found in serum samples sampled 6 h or later postinfection (Trial 2). Until now, no reports on whether NetB toxin present in the intestine can penetrate the epithelium barrier to reach circulation have been published. Løvland and Kaldhusdal (1999) isolated *C. perfringens* from the liver of NE-afflicted chickens exhibiting cholangitis at the slaughterhouse, indicating systemic exposure of intestine-origin *C. perfringens* or their toxins via the portal system to the hepatocyte or bile ducts (Moran 2014). Our study supports earlier speculations by Moran (2014) that NetB toxin originating from the gut lumen could penetrate the gut barrier for systemic circulation.

It is clear from this study that native NetB toxin was detected in the digesta or fecal droppings sampled 6 h or later postinfection, and the toxin concentration plateaued 24 h postinfection (Trial 2). In addition, 3 out of 8 fecal droppings originating from the Georgia NE chicken model had detectable concentrations of NetB toxin. Furthermore, the concentration of NetB toxin ranged from 2.9 to 3.1 ng per g of wet feces in Trial 3, which is far lower than the values (0.19–17.6 ng per g of wet feces) found in Trial 2. This difference might be from the difference in the *C. perfringens* strains used in the NE model and the sampling time point after *C. perfringens* infection. We reported a definite difference in the capability of *netB*-positive *C. perfringens* strains in secreting NetB toxin (Lee et al., 2020). In addition, it is well known that NE-specific clinical lesions in broiler chickens gradually disappeared, emphasizing the importance of earlier NE scoring or tissue sampling in experimental NE disease models (Lee et al., 2011).

Of particular interest is the significant correlation between NE lesions and the presence of NetB toxin in the gut digesta (Figure 1D). Considering the role of NetB toxin (i.e., pore-forming) in NE pathogenesis, it seems that the observed association is not more significant than expected. The number of birds (*n* = 22) sampled during the course of NE development and dominant clinical birds with NE lesions being 8 out of 12 birds might cause skewed or irrelevant, however moderate, associations between 2 factors. A clear explanation needs to be ascertained using more biological samples from broiler chickens with varying degrees of NE lesions.

Keyburn et al. (2008) found that purified NetB protein ranging from 1.89 to 242.4 nM (calculated from 8 µg to 62.5 ng/well in a 24-well format) induced the cytotoxicity effect on chicken hepatocellular carcinoma cell line (LMH cells). In line with the study by Keyburn et al. (2008), Savva et al. (2013) also observed that purified NetB induced morphological damage on LMH cells. However, Savva et al. (2013) used much higher NetB concentrations ranging from 62.5 to 4,000 nM (calculated from approximately 412.5 ng to 26.4 µg/well in 96-well format) in the cytotoxicity assay with a median cytotoxic dose of 800 nM. In our study, the approximate concentration of NetB toxin in the gut digesta was estimated to be 2.23 nM (calculated from 73.5 ng per intestine). Thus, the preliminary study showed that these cytotoxic concentrations used in in vitro studies (Keyburn et al., 2008) are likely to be biologically relevant to NE development in vivo.

In conclusion, we attempted to quantify the presence of native NetB toxin in biological samples of NE chickens using NetB-specific capture ELISA. This is the first report demonstrating that native NetB toxin was present in various biological samples obtained from NE-afflicted broilers. Currently, we are conducting a study to monitor or quantify the presence of NetB toxin in biological samples collected from broiler production farms with a history of none to severe NE.

## DISCLOSURES

No potential conflict of interest was reported by the authors.
